# Eurycomanone Blocks TGF-β1-Induced Epithelial-to-Mesenchymal Transition, Migration, and Invasion Pathways in Human Non-Small Cell Lung Cancer Cells by Targeting Smad and Non-Smad Signaling

**DOI:** 10.3390/ijms26157120

**Published:** 2025-07-23

**Authors:** Pratchayanon Soddaen, Kongthawat Chairatvit, Pornsiri Pitchakarn, Tanongsak Laowanitwattana, Arisa Imsumran, Ariyaphong Wongnoppavich

**Affiliations:** 1 Graduate/M.Sc. Program in Biochemistry, Faculty of Medicine, Chiang Mai University, Chiang Mai 50200, Thailand; pratchayanon_s@hotmail.com; 2 Department of Biochemistry, Faculty of Medicine, Chiang Mai University, Chiang Mai 50200, Thailand; pornsiri.p@cmu.ac.th (P.P.); tanongsak.l@cmu.ac.th (T.L.); arisa.bonness@cmu.ac.th (A.I.); 3 Department of Oral Biology, Faculty of Dentistry, Mahidol University, Bangkok 10400, Thailand; kongthawat.cha@mahidol.ac.th

**Keywords:** eurycomalactone, eurycomanone, invasion, epithelial-to-mesenchymal transition, non-small cell lung cancer

## Abstract

Non-small cell lung cancer (NSCLC) is a predominant form of lung cancer that is often diagnosed at an advanced metastatic stage. The processes of cancer cell migration and invasion involve epithelial-to-mesenchymal transition (EMT), which is crucial for metastasis. Targeting cancer aggressiveness with effective plant compounds has gained attention as a potential adjuvant therapy. Eurycomanone (ECN), a bioactive quassinoid found in the root of *Eurycoma longifolia* Jack, has demonstrated anti-cancer activity against various carcinoma cell lines, including human NSCLC cells. This study aimed to investigate the in vitro effects of ECN on the migration and invasion of human NSCLC cells and to elucidate the mechanisms by which ECN modulates the EMT in these cells. Non-toxic doses (≤IC20) of ECN were determined using the MTT assay on two human NSCLC cell lines: A549 and Calu-1. The results from wound healing and transwell migration assays indicated that ECN significantly suppressed the migration of both TGF-β1-induced A549 and Calu-1 cells. ECN exhibited a strong anti-invasive effect, as its non-toxic doses significantly suppressed the TGF-β1-induced invasion of NSCLC cells through Matrigel and decreased the secretion of MMP-2 from these cancer cells. Furthermore, ECN could affect the TGF-β1-induced EMT process in various ways in NSCLC cells. In TGF-β1-induced A549 cells, ECN significantly restored the expression of E-cadherin by inhibiting the Akt signaling pathway. Conversely, in Calu-1, ECN reduced the aggressive phenotype by decreasing the expression of the mesenchymal protein N-cadherin and inhibiting the TGF-β1/Smad pathway. In conclusion, this study demonstrated the anti-invasive activity of eurycomanone from *E. longifolia* Jack in human NSCLC cells and provided insights into its mechanism of action by suppressing the effects of TGF-β1 signaling on the EMT program. These findings offer scientific evidence to support the potential of ECN as an alternative therapy for metastatic NSCLC.

## 1. Introduction

Lung cancer is still a major cause of cancer-related mortality and is most often diagnosed at a late stage. Highly progressive cancer cells are likely to spread to more than one area in the lung, the fluid surrounding the lung, or distant parts of the body, which makes it hard to cure [1]. Non-small cell lung cancer (NSCLC) is the most common type of lung cancer, accounting for approximately 85% of all lung cancer cases [2]. More than half of diagnosed NSCLC cases are identified at an advanced stage, and these patients typically have a low five-year survival rate [3,4]. Cancer invasion is an early step in the metastatic process, where invasive cancer cells disrupt their basement membrane. These cells then migrate into the neighboring stromal tissue before entering the circulatory system to disseminate. The loss of cell–cell contacts and cell-basement membrane interaction occurs due to a decrease in cell adhesion molecules and changes in the cytoskeleton, which allows them to detach from the primary site. The cleavage of extracellular matrix (ECM) components by protease enzymes such as matrix metalloproteinases (MMPs) is necessary for cancer cell migration and invasion through the degraded ECM [5]. Moreover, the epithelial-to-mesenchymal transition (EMT) plays a crucial role in promoting cancer invasion. During EMT, cancer cells lose their epithelial features due to a reduction in cell–cell adhesion molecules like E-cadherin, and they acquire mesenchymal phenotypes characterized by increased levels of proteins such as N-cadherin, vimentin, and fibronectin [6]. As cancer progresses, various cytokines and growth factors, including transforming growth factor-β1 (TGF-β1) from the tumor environment, trigger a series of intracellular signaling pathways that subsequently promote EMT [7]. The enhanced invasiveness capabilities of EMT ultimately contribute to cancer metastasis. Therefore, therapeutic strategies aimed at inhibiting the invasive properties of cancer cells may be useful in preventing the spread of organ-confined cancers in patients at high risk of developing metastases, and in targeting existing metastatic cells in patients with more advanced disease [8].

Strategies for inhibiting the EMT pathway to control cancer metastasis include blocking EMT-inducing signaling molecules from the microenvironment, inhibiting downstream EMT-signal transduction pathways, and targeting proteins associated with the mesenchymal phenotype. Recently, effective natural plant compounds and their extract have been actively investigated as a potential adjuvant therapy for cancer treatment by targeting EMT. For example, paeonol from *Paeonia suffruticosa* has been found to inhibit cell motility, EMT, and MMPs in the NSCLC cells stimulated by TNF-α or IL-6. This compound also inhibited NF-κB and STAT3 signaling pathways in NSCLC [9]. Furthermore, resveratrol, which is widely found in grapes and other plants, has been shown to reduce lung cancer cell invasion and metastasis. It increased E-cadherin and suppressed the expression of vimentin and fibronectin through the downregulation of EMT-inducing transcription factors, SNAI1 and SLUG [10]. Thus, identifying chemopreventive agents that target EMT is an attractive approach to prevent or reduce the invasive capacities, including migration and invasion of cancer cells.

*Eurycoma longifolia* Jack., commonly known as “Tongkat Ali” in Malaysia, “Pasak Bumi” in Indonesia, or “Pla lai phueak” in Thailand, is a popular traditional herbal medicine in Southeast Asia. The root of *E. longifolia* is traditionally used to treat sexual dysfunction, malaria, diabetes, and anxiety, as well as serving as an appetite stimulant and health supplement [11,12]. The root extract is known to be rich in many bioactive compounds, including quassinoids, alkaloids, triterpenes of the tirucallane type, squalene derivatives, and bioactive steroids [11,12]. Several studies have reported various bioactivities of quassinoid compounds, such as antimalarial, antimicrobial, anti-inflammatory, antidiabetic, and potent anti-cancer properties [12,13]. Eurycomalactone (ECL) and eurycomanone (ECN) are two major quassinoids found in the root extract of *E. longifolia* for several compelling reasons, primarily based on their abundance, their role as chemical markers for standardization, and their potent biological activities [12,13]. The standardized water extract requires ECN levels to be consistently present at 0.80–1.50% *w*/*v* [14]. The quantitative yield of ECN extracted from the roots was around 1.61 mg/g dry weight using the optimized natural deep eutectic solvent and microwave-assisted extraction [15]. Both compounds exhibit anti-cancer activity, including strong cytotoxic effects against various carcinoma cell lines, such as human NSCLC cells [13,14,15,16,17]. They could induce apoptotic cell death in cancer cells and inactivate the NF-κB signaling pathway [18,19]. Furthermore, sub- or non-toxic concentrations of these compounds may serve as chemo/radio sensitizing agents to enhance the efficacy of cancer treatments through the induction of cancer cell death [20]. Nonetheless, we hypothesized that *E. longifolia* has an anti-invasive effect on human cancer cells. This study aims to explore the anti-invasive potential of purified compounds (ECL and ECN) against human NSCLC cells. Our goal is to generate mechanistic insights that support the ethnobotanical and therapeutic claims associated with *E. longifolia*. Our study is the first to demonstrate that ECN inhibits cell migration, MMP-2 secretion, and invasion of human NSCLC cell lines, providing insights into the underlying mechanisms through regulating the EMT program in cancer cells.

## 2. Results

### 2.1. ECL and ECN Are Cytotoxic Against Human NSCLC Cells

**Figure 1:**
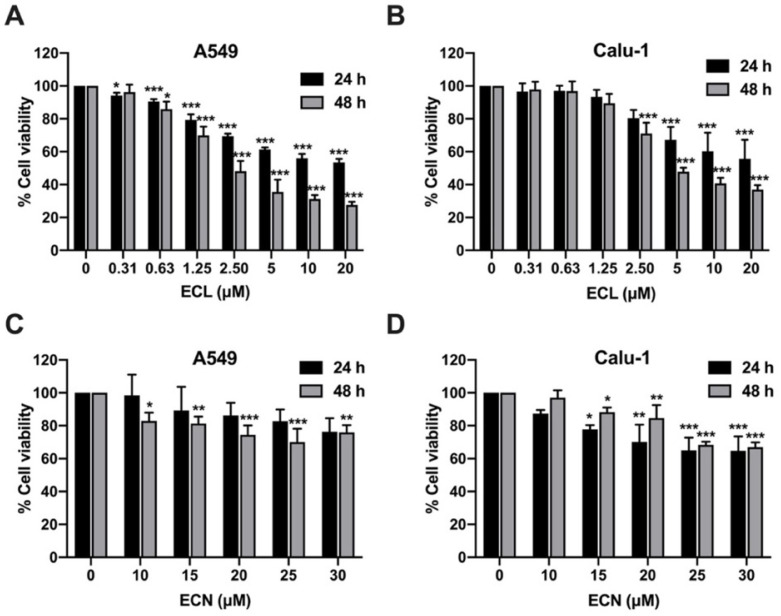
Cytotoxic Effects of ECL and ECN on Human NSCLC Cells. Using an MTT assay. The percentages of cell viability for A549 and Calu-1 were assessed after treatment with varying concentrations of (**A**,**B**) ECL (0–20 μM) or (**C**,**D**) ECN (0–30 μM) for 24 and 48 h. All data are presented as the mean ± SD of three independent experiments. * *p* < 0.05, ** *p* < 0.01, *** *p* < 0.001 versus the non-treated control.

Eurycomalactone (ECL) and eurycomanone (ECN) are significant bioactive compounds found in *E. longifolia* and have previously demonstrated anti-cancer activities [13]. We conducted an MTT assay to evaluate the cytotoxic effects of ECL and ECN on human NSCLC cells, A549 and Calu-1 cells. Both types of cancer cells were incubated with varying concentrations of ECL (0–20 μM) or ECN (0–30 μM) for 24 and 48 h. As shown in Figure 1, treatment with ECL significantly reduced the viability of both A549 and Calu-1 cells in a dose- and time-dependent manner. In contrast, ECN noticeably exhibited lower cytotoxicity compared to ECL in both NSCLC cells. In addition, ECL demonstrated a more potent effect on cell viability against A549 than on Calu-1 cells. For Calu-1 cell viability, a higher concentration of ECN was required to achieve a reduction at 48 h than at 24 h. The 20% and 50% inhibitory concentrations of cell viability (IC20 and IC50) for ECL and ECN after 24 h and 48 h of treatment in both A549 and Calu-1 cells are shown in Table 1. ECL showed lower IC20 and IC50 values than ECN, suggesting that ECL’s cytotoxic effects are more potent than ECN in both NSCLC cell lines. Therefore, we chose non-toxic concentrations (≤IC20) of ECL and ECN on A549 and Calu-1 cells for further investigation into their anti-migration and anti-invasion properties.

### 2.2. ECN, but Not ECL, Inhibits TGF-β1-Induced Migration of NSCLC Cells and MMP-2 Secretion

**Figure 2:**
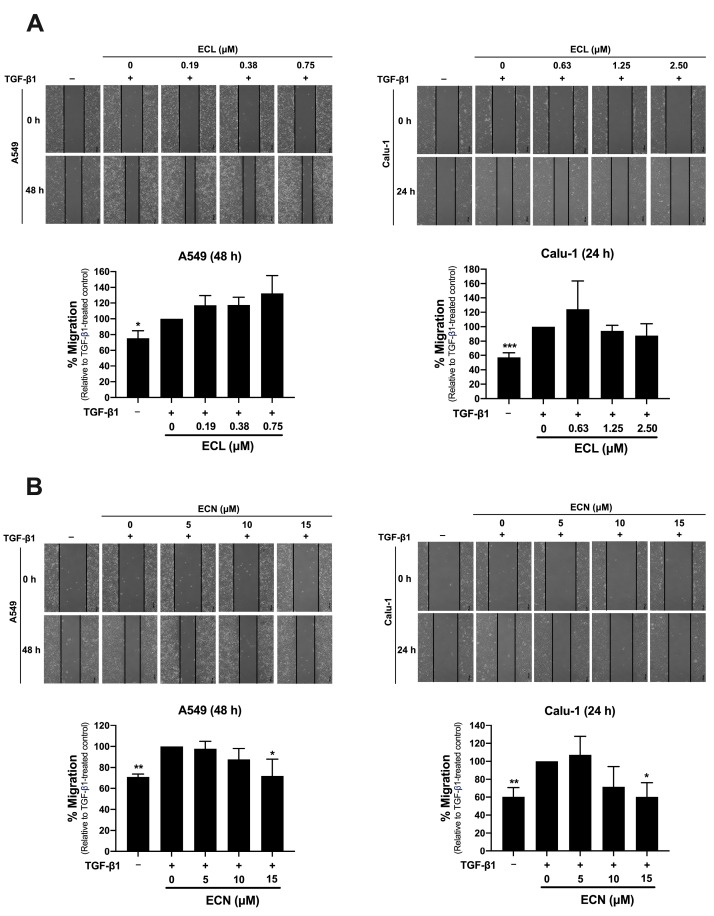
Effect of ECL and ECN on TGF-β1-Induced Migration in Human NSCLC Cells Using a wound healing assay, the migration of A549 or Calu-1 cells was assessed following co-treatment with or without (**A**) TGF-β1 (10 ng/mL) + ECL or (**B**) TGF-β1 (10 ng/mL) + ECN. The percentages of cell migration were normalized to the TGF-β1-treated control group. All data are presented as the mean ± SD of three independent experiments. * *p* < 0.05, ** *p* < 0.01, *** *p* < 0.001 versus the TGF-β1-treated control group. The scale bar = 200 μm.

To evaluate whether the non-toxic concentrations of ECL and ECN can inhibit the TGF-β1-induced migration of NSCLC cells, we conducted a wound-healing assay. Due to differences in cell aggressiveness, A549 cells required a longer incubation time for migration (48 h) compared to Calu-1 cells (24 h). After treatment with TGF-β1, migration rates for both A549 and Calu-1 cells significantly increased compared to the control group untreated with TGF-β1. Interestingly, treatment with ECN at 15 μM significantly decreased TGF-β1-stimulated migration by 30% and 40% of A549 and Calu-1, respectively, when compared to the TGF-β1-treated control group (Figure 2B). In contrast, ECL did not inhibit the TGF-β1-stimulated migration in either A549 or Calu-1 cells (Figure 2A).

**Figure 3:**
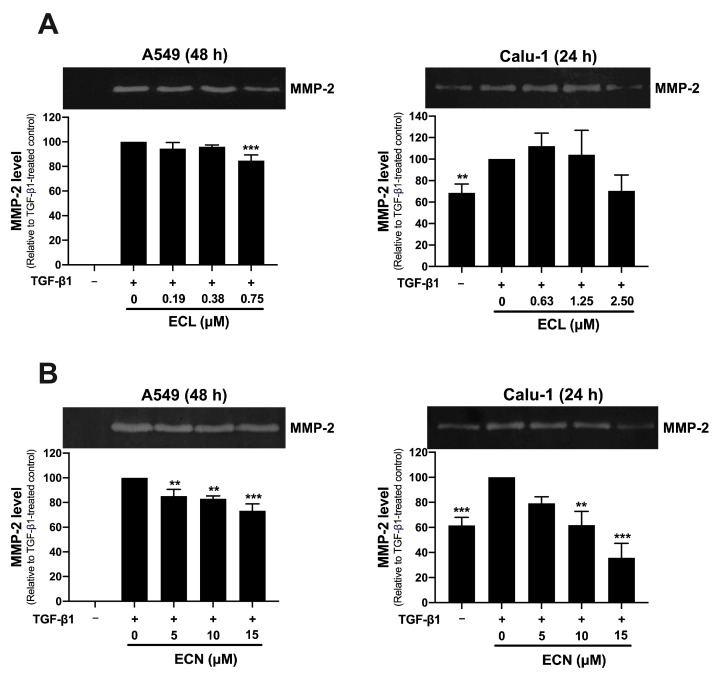
Effects of ECL and ECN on TGF-β1-induced MMP-2 secretion in human NSCLC Cells. The zymogram of MMP-2 secretion and quantification of MMP-2 secretion level in A549 cells or Calu-1 cells after the co-treatment with or without (**A**) TGF-β1 (10 ng/mL) + ECL or (**B**) TGF-β1 (10 ng/mL) + ECN for 24 or 48 h. The intensity of the clear band was normalized against the TGF-β1-treated control group. All data are presented as the mean ± SD of three independent experiments. ** *p* < 0.01, *** *p* < 0.001 versus the TGF-β1-treated control group.

MMP-2 is a protease that can be secreted by cancer cells to degrade collagen components in the ECM, which facilitates cancer invasion. We evaluated the effect of ECL and ECN on the TGF-β1-induced secretion of MMP-2 from NSCLC cells using a gelatin zymography assay. The results, indicated by clear bands on the stained gel, demonstrate the level of MMP-2 secretion, showing distinct zones of gelatinolytic activity. Our findings revealed that TGF-β1 significantly increased MMP-2 secretion in both cell types studied, with the effect being particularly pronounced in A549 cells compared to the non-TGF-β1 treated group. The treatment of ECL in A549 cells at 0.75 μM and in Calu-1 cells at 2.50 μM led to a significant decrease in MMP-2 secretion by 15% and 30%, respectively, when compared to the TGF-β1 treated control (Figure 3A). Interestingly, the treatment with ECN in A549 cells at concentrations of 5, 10, and 15 μM significantly inhibited TGF-β1-induced MMP-2 secretion by approximately 20%. In Calu-1 cells, ECN treatment at 10 and 15 μM resulted in significant decreases of 40% and 70%, respectively (Figure 3B). These results suggest that only ECN exhibits a strong anti-invasive effect through significant inhibition of TGF-β1-induced migration and MMP-2 secretion in NSCLC cells. Consequently, ECN was selected for further investigation into its anti-invasive activity in subsequent experiments.

### 2.3. ECN Suppresses TGF-β1-Induced Migration and Invasion of NSCLC Cells

**Figure 4:**
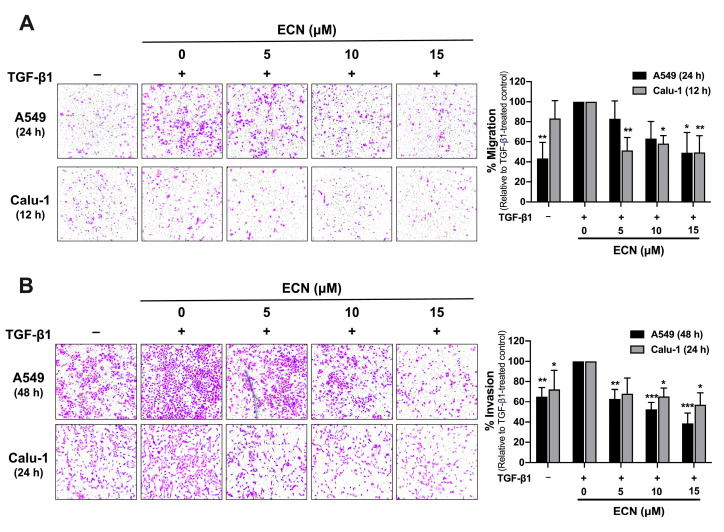
Effect of ECN on TGF-β1-induced migration and invasion in human NSCLC cells. The representative images (10× magnification) and quantification of A549 and Calu-1 cells were assessed for (**A**) cell migration and (**B**) invasion using transwell migration and invasion assays, respectively. Cells were co-treated with TGF-β1 (10 ng/mL) and ECN for 12, 24, or 48 h. The percentages of migration or invasion were normalized to the TGF-β1-treated control group. All data are presented as the mean ± SD of three independent experiments. * *p* < 0.05, ** *p* < 0.01, *** *p* < 0.001 versus the TGF-β1-treated control group.

Cancer cell invasion through the ECM is accompanied by the secretion of proteases and an increase in cellular motility. To investigate the effect of ECN on TGF-β1-induced cell migration, we conducted a transwell migration assay. As shown in Figure 4A, treatment with TGF-β1 significantly increased the migration of both A549 and Calu-1 cells compared to the group that did not receive TGF-β1. Conversely, treatment with ECN at 15 μM significantly reduced the migration of A549 cells by 50%. Moreover, all doses of ECN significantly inhibited the TGF-β1-induced migration of Calu-1 cells, resulting in a decrease of approximately 50% compared to the TGF-β1-treated control. To further assess the impact of ECN on TGF-β1-stimulated NSCLC cell invasion, we employed a Matrigel transwell invasion assay. The results showed in Figure 4B that TGF-β1 highly increased the invasion of both A549 and Calu-1 cells through Matrigel when compared to the non-TGF-β1 treated group. Treatment with ECN at concentrations of 5, 10, and 15 μM for 48 h markedly reduced the invasion of A549 cells by 40%, 50%, and 60%, respectively. Likewise, ECN at concentrations of 10 and 15 μM significantly inhibited the TGF-β1-induced invasion of Calu-1 cells by approximately 40% compared to the control group treated with TGF-β1. Taken together, these findings indicate that ECN suppresses TGF-β1-induced NSCLC cell invasion by decreasing the secretion of MMP-2 and blocking the migration of NSCLC cells.

### 2.4. ECN Suppresses TGF-β1-Induced Epithelial-to-Mesenchymal Transition of NSCLC Cells

**Figure 5:**
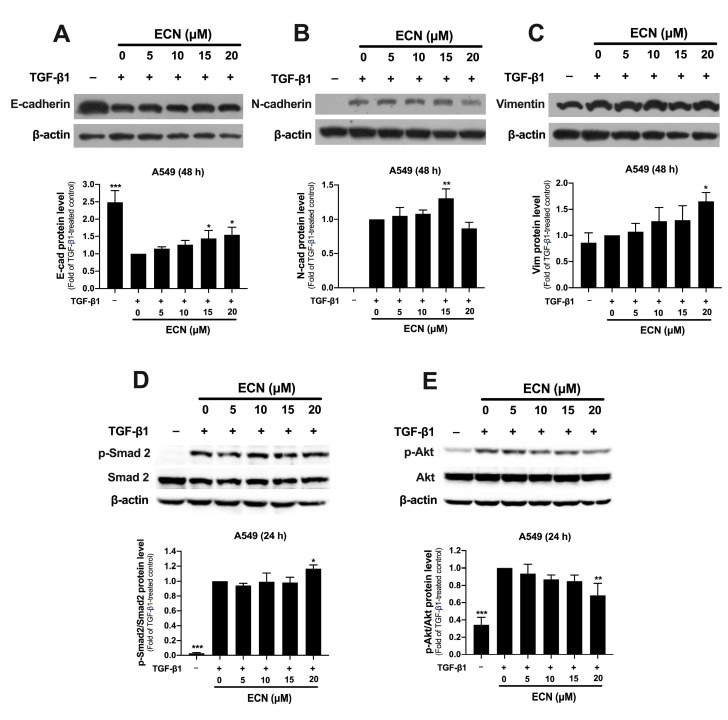
Effect of ECN on expression of EMT-associated proteins and TGF-β1 signaling proteins in TGF-β1-treated A549 cells. Representative immunoblots with quantification of (**A**) E-cadherin, (**B**) N-cadherin, (**C**) vimentin, (**D**) p-Smad2 and total Smad2, and (**E**) p-Akt and total Akt expression in A549 cells after co-treatment with or without TGF-β1 (10 ng/mL) and ECN for 24 or 48 h. β-actin was used as an internal control. The levels of the targeted proteins were normalized to β-actin and are represented as relative to the TGF-β1-treated control group. All data are presented as the mean ± SD of three independent experiments. * *p* < 0.05, ** *p* < 0.01, *** *p* < 0.001 versus the TGF-β1-treated control group.

TGF-β1 plays a significant role in cancer progression by inducing EMT, which leads to increased invasiveness of cancer cells. We assessed the expression of EMT marker proteins in A549 cells treated with or without TGF-β1 and ECN for 24 or 48 h, using Western blotting. As shown in Figure 5A–C, TGF-β1 noticeably downregulated the expression of E-cadherin, but upregulated that of N-cadherin and vimentin compared to the untreated control group. In contrast, treatment with ECN upregulated E-cadherin expression in a dose-dependent manner relative to the TGF-β1-treated group. Notably, ECN at a concentration of 15 μM significantly enhanced TGF-β1-induced N-cadherin expression, although the highest concentration of ECN did not produce any effect. Additionally, ECN treatment did not inhibit but likely increased the TGF-β1-induced vimentin expression compared to the TGF-β1-treated control.

Next, we examined the effect of ECN on the TGF-β1 signaling pathway in A549 cells. As anticipated, TGF-β1 activated Smad and Akt signaling, significantly increasing the ratio of phosphorylated Smad2 (p-Smad2) to total Smad2 and phosphorylated Akt (p-Akt) to total Akt when compared to the untreated group (Figure 5D,E). Interestingly, treatment with ECN at 20 μM significantly reduced only the ratio of p-Akt to total Akt (Figure 5E) while not affecting Smad signaling in TGF-β1-treated A549 cells. These findings suggest that ECN may help restore the epithelial phenotype in A549 cells undergoing EMT by blocking the non-Smad dependent pathway of TGF-β1 signaling.

### 2.5. ECN Reduces the Aggressive Phenotype of TGF-β1-Stimulated Calu-1 Cells

**Figure 6:**
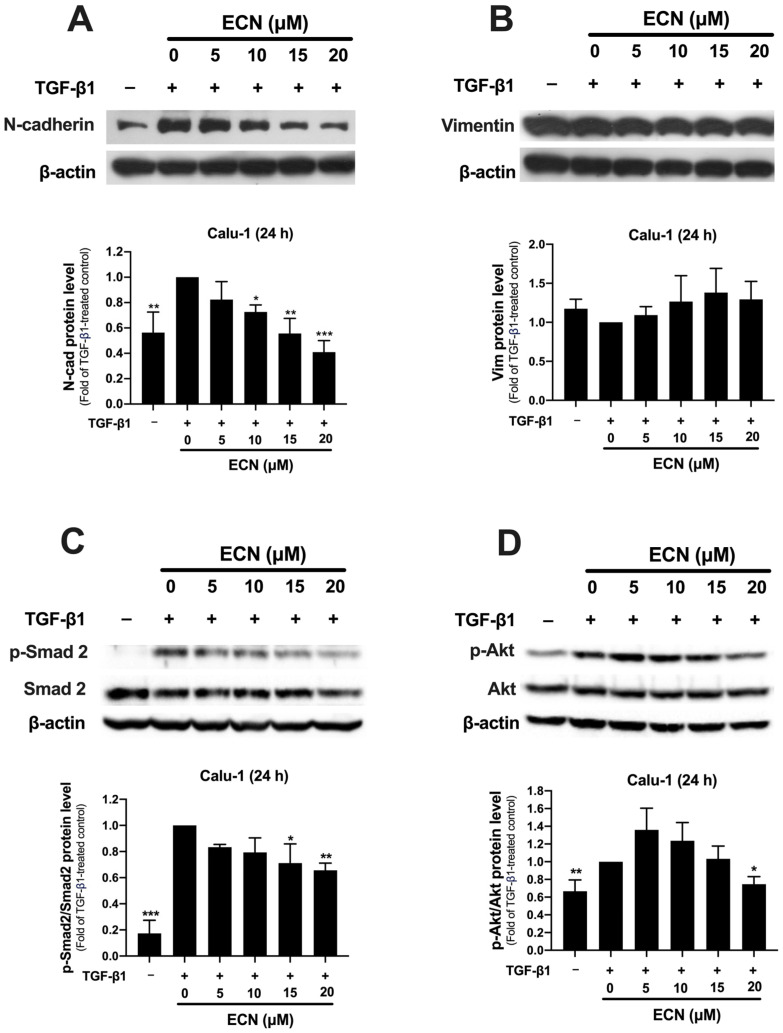
Effects of ECN on EMT-associated protein and TGF-β1 signaling protein expression in TGF-β1-treated Calu-1 cells determined by Western blotting. (**A**) The representative immunoblots with quantification of N-cadherin, (**B**) vimentin, (**C**) p-Smad2 and total Smad2 and (**D**) p-Akt and total Akt expression of Calu-1 cells after co-treated with or without TGF-β1 (10 ng/mL) and ECN for 24 h. β-actin was used as an internal control. The band of targeted protein was normalized with β-actin and represents as relative to the TGF-β1-treated control group. All data are presented as the mean ± SD of three independent experiments. * *p* < 0.05, ** *p* < 0.01, *** *p* < 0.001 versus the TGF-β1-treated control group.

We confirmed the effect of ECN on the induction of EMT by TGF-β1 in the highly invasive NSCLC cells, Calu-1. These cells, having a mesenchymal-like phenotype, showed no expression of E-cadherin, but constitutively expressed N-cadherin and vimentin (Figure 6A,B). Notably, ECN did not induce E-cadherin expression in Calu-1 cells. Additionally, TGF-β1 highly increased the expression of N-cadherin, but this effect was significantly reduced with ECN treatment in a dose-dependent manner. However, there were no significant changes in vimentin expression in TGF-β1-treated groups, whether or not they were treated with ECN. Consistent with previous results, TGF-β1 significantly increased the ratio of p-Smad2 to Smad2 and p-Akt to Akt in Calu-1 cells when compared to the untreated group. In contrast to the previous findings in A549, the treatment of Calu-1 cells with ECN significantly suppressed the ratio of p-Smad2 to Smad2 in a dose-dependent manner (Figure 6C). ECN at a concentration of 20 μM was the only treatment significantly decreasing the ratio of p-Akt to Akt (Figure 6D). Therefore, it is possible that in the highly invasive cancer cells, such as Calu-1, ECN could inhibit both Smad-dependent and independent signaling pathways induced by TGF-β1. This inhibition may lead to a less invasive phenotype of the cancer cells, characterized by reduced N-cadherin expression due to ECN treatment.

## 3. Discussion

Non-small cell lung cancer (NSCLC) is the most common type of lung cancer [2]. More than half of diagnosed NSCLC cases are typically found at the advanced stage of metastatic cancer [3], which is often associated with treatment failure and increased mortality in patients with malignant tumors. Invasion plays a crucial role in initiating cancer metastasis. The development of chemopreventive agents is needed to prevent or reduce the invasive capabilities of cancer cells, thereby effectively attenuating metastasis. Recently, there has been a growing interest in using medicinal plant compounds as a potential adjuvant therapy to inhibit cancer progression.

*Eurycoma longifolia* Jack contains major quassinoids known as ECL and ECN, which have demonstrated anti-cancer activity. By focusing on purified compounds, the research aims to generate mechanistic insights that support the ethnobotanical and therapeutic claims associated with *E. longifolia* root, while recognizing the limitations of extrapolating in vitro results to physiological contexts. While in vitro models may not accurately represent ECN’s in vivo activity due to poor absorption, ECN is a highly polar compound that remains stable across various pH levels, in plasma, and in liver microsomes across species, including humans [14]. It exhibits low plasma protein binding (30% in rats, 38.9% in humans), which means a significant fraction of unbound ECN (61.1–78.4%) is freely available to target sites. This suggests that overcoming the permeability barrier through novel delivery systems could allow the compound to effectively reach its targets. Mechanistic studies are then needed to understand its cellular effects, with confidence that it will not degrade rapidly or bind extensively in biological systems.

Both ECL and ECN exhibit cytotoxic effects against various cancer cell lines, including NSCLC cells [16], induce apoptotic cancer cell death [17,18], and inhibit the NF-κB signaling pathway [18,19]. In our study, the IC20 and IC50 values for NSCLC cell viability of ECL were significantly lower than those of ECN by more than ten times. This suggests that ECL has a more potent cytotoxic effect than ECN in both A549 and Calu-1 cells. Consistent with previous studies, among the isolated quassinoids from the *E. longifolia* roots, ECL showed the strongest anti-cancer activity against all tested cancer cell lines [13,16]. Moreover, the anti-cancer effects of ECL and ECN are attributed to their inhibition of the NF-κB signaling pathway, which is involved in cancer cell survival and apoptosis [18,19]. ECL is a more effective NF-κB inhibitor than ECN in TNF-α-induced HEK-293/NF-κB-luc cells [21]. Thus, the enhanced cytotoxic effect of ECL in NSCLC cells can be linked to its strong inhibitory activity against the NF-κB pathway. In addition to their anti-cancer properties, previous studies have demonstrated that 14,15β-dihydroxyklaineanone from *E. longifolia* Jack exhibits anti-metastatic activity [22]. Several quassinoids from other plants also show anti-migration and anti-invasion effects against various cancer cells by regulating the expression of EMT-related proteins and the signaling pathways that induce EMT [23,24,25]. In this study, we aimed to investigate the anti-cancer invasive activities of ECL and ECN at non-toxic concentrations (≤IC20) and elucidate the mechanism involved in the EMT process.

TGF-β1 is a crucial cytokine that plays a pivotal role in the progression of cancer [7]. It can reduce cancer cell adhesion, increase cell motility, and promote the production of ECM-degrading enzymes, particularly MMP-2 and MMP-9 [26]. These effects enhance the invasive behavior in various types of cancer, including lung cancer [27]. Inhibiting TGF-β1-induced MMP-2 expression and cell migration has been shown to effectively decrease invasion and metastasis in lung carcinoma and melanoma [28]. The present study is the first to demonstrate the anti-invasive effects of ECL and ECN on NSCLC cells stimulated by TGF-β1. Notably, treatment with non-toxic doses of ECN significantly inhibited both TGF-β1-induced cell migration and MMP-2 secretion in NSCLC cells. In contrast, ECL only inhibited MMP-2 secretion without affecting TGF-β1-induced cell migration. The higher cytotoxicity of ECL (with a very low IC20) compared to ECN against NSCLC cells suggests that ECL might be more effective in inducing cell death. The Matrigel transwell invasion assay confirmed that ECN significantly reduced the invasion of TGF-β1-treated A549 and Calu-1 cells. These findings suggest that ECN inhibits cell invasion by suppressing MMP-2 secretion and migration in NSCLC cells.

EMT is a well-known process in cancer progression and metastasis, characterized by the transformation of epithelial cells into a mesenchymal phenotype [29]. This transition not only reduces cell–cell adhesion and alters the cellular cytoskeleton [30] but also enhances the degradation of the ECM [31]. During EMT, the expression of epithelial cell adhesion molecules, such as E-cadherin, is decreased, while mesenchymal proteins like N-cadherin and vimentin are upregulated [32]. Thus, restoring E-cadherin expression and inhibiting the expression of N-cadherin/vimentin could potentially prevent the initiation of EMT, thereby reducing the migration and invasiveness of malignant tumors. In our study, we employed a model of TGF-β1-induced EMT in primary-origin lung cancer cell lines. Unlike Calu-1 cells, A549 displayed a more epithelial phenotype with higher levels of endogenous E-cadherin expression. As expected, treatment with TGF-β1 caused EMT in A549 cells, resulting in decreased E-cadherin expression and increased levels of N-cadherin and vimentin. It is important to note that loss of E-cadherin is a key event in EMT, which is well-known for promoting cell migration and invasion. During this transition, the induction of specific mesenchymal markers, such as vimentin, is also increased [33]. Furthermore, the loss of E-cadherin has been shown to activate MMP-2 and MMP-9. This activation leads to the degradation of the basement membrane, which facilitates the initial invasion of tumor cells in squamous cell carcinoma [34]. Therefore, the loss of cell–cell adhesion significantly contributes to the enhanced invasive behavior of cancer cells.

In the present study, we demonstrate that the treatment with ECN significantly upregulated the expression of E-cadherin, and to a lesser extent, that of N-cadherin and vimentin. Interestingly, ECN still suppressed the migration and invasion of TGF-β1-treated A549 cells, despite the slight increase in mesenchymal proteins. This suggests that E-cadherin may counteract the effects of N-cadherin and vimentin expression. Similar to our findings, the induction of E-cadherin expression by trichostatin A and valproic acid was sufficient to inhibit cell migration and invasion of cholangiocellular carcinoma cells, even though it also caused an increase in the mesenchymal protein vimentin [35]. These results emphasize the potentially critical role of E-cadherin in preventing the progression of epithelial cancer to a metastatic state. Previous studies have shown that the induction of E-cadherin expression led to the suppression of MMP-2 activity in human colon carcinoma cells [36], reduced cellular invasion in prostatic adenocarcinoma [37], and down-regulated the expression of various types of MMPs in highly invasive bronchial tumor cells [38]. Increased E-cadherin expression may enhance cell–cell adhesion more effectively than N-cadherin, which is important for maintaining the epithelial phenotype and restraining cell migration [39,40]. Therefore, our results suggest that the restoration of E-cadherin through ECN is a potential modulator in controlling cancer cell migration, MMP-2 secretion, and invasion by preventing the EMT process in TGF-β1-stimulated A549 cells. Finally, ECN helps preserve the epithelial phenotype of A549 cells, leading to decreased invasion of TGF-β1-induced A549 cells.

Calu-1, unlike those in A549, are mesenchymal-like cancer cell lines that have higher invasive capacities and increased expression of N-cadherin. The invasive cancer cells typically exhibit high levels of mesenchymal proteins, such as N-cadherin and vimentin [41,42]. Increased N-cadherin expression leads to weaker adherent junctions, which in turn promote cellular motility, MMP-9 induction, and invasion in some carcinoma cell lines [43,44]. Moreover, upregulation of vimentin is linked to a migratory phenotype in cancer cells [45]. Therefore, targeting these proteins may provide a strategy to eliminate or reduce the aggressive behavior of metastatic cancer cells in patients with advanced disease [46]. In our study, we found that TGF-β1 can induce the expression of N-cadherin in Calu-1 cells, but it does not affect vimentin levels. Interestingly, treatment with ECN significantly downregulated the expression of N-cadherin. It has been shown that knocking down N-cadherin expression inhibited the activities of MMP-2 and MMP-9, leading to reduced cell invasion in both primary and metastatic melanoma cell lines [47]. Thus, ECN may effectively reduce the aggressive phenotype of TGF-β1-treated Calu-1 cells by downregulating N-cadherin expression, resulting in reduced migration and invasion capabilities.

TGF-β1 activates EMT through various downstream signaling pathways, including both Smad-dependent (TGF-β/Smad) and non-Smad pathways (such as PI3K/Akt). These pathways promote the migratory and invasive capabilities of cancer cells [29,32]. As expected, TGF-β1 increased the phosphorylation of Smad 2 and Akt, indicating the activation of Smad and non-Smad signaling pathways in both A549 and Calu-1 cells. The TGF-β1 signaling pathway is associated with the occurrence of EMT, which leads to a significant downregulation in E-cadherin in A549 cells and a predominant upregulation of N-cadherin in Calu-1 cells. Activation of the Akt pathway has been shown to induce EMT, leading to a substantial reduction in E-cadherin expression in cancer cells [48,49]. In contrast, inhibiting Akt signaling can prevent EMT by promoting E-cadherin expression while simultaneously decreasing the expression of mesenchymal proteins [50,51]. The increased E-cadherin levels may help maintain binding with the catenin complex [37], thereby preventing the activation of additional EMT-inducing signaling pathways, such as PI3K/Akt [39]. Furthermore, inhibiting Smad signaling can lead to the downregulation of mesenchymal markers, including N-cadherin [52,53]. The present data demonstrated that ECN decreased the p-Akt/Akt ratio in a dose-dependent manner in A549 cells, while in Calu-1 cells, it decreased the p-Smad2/Smad2 ratio. This indicates that ECN exerts different effects in different cell types, suppressing Akt signaling in A549 cells and Smad signaling in Calu-1 cells.

Our data reveal that ECN exhibits a biphasic effect on Akt phosphorylation in Calu-1 cells, with low concentrations increasing p-Akt levels and higher concentrations decreasing them. This phenomenon is consistent with established feedback mechanisms in the PI3K/Akt pathway [54]. Low doses of mTOR kinase inhibitors can lead to partial inhibition, relieving negative feedback and transiently enhancing Akt phosphorylation. In contrast, higher doses effectively suppress upstream signals. Furthermore, the presence of TGF-β1, an Akt activator, suggests that low doses of ECN may enhance TGF-β1-induced signaling. Similar findings have been reported in a 2023 study by Zhong et al. [55], which noted that ECN’s effects on Akt signaling are dose- and context-dependent, enhancing Akt activation at 0.2–5 μM to promote osteogenic differentiation. Overall, this biphasic response highlights the complex regulation of Akt phosphorylation by eurycomanone, which is influenced by concentration, cellular context, and external stimuli.

In conclusion, eurycomanone (ECN), a bioactive quassinoid compound from *E. longifolia* Jack, effectively suppressed the epithelial–mesenchymal transition (EMT) induced by TGF-β1. This suppression was achieved through the upregulation of E-cadherin and downregulation of N-cadherin expression. Treatment with ECN led to a significant reduction in cell migration, secretion of MMP-2, and invasion in both low-invasive A549 and high-invasive Calu-1 NSCLC cells. The inhibition of invasive properties in NSCLC cells by ECN was linked to the disruption of the TGF-β1/Smad and/or Akt signaling pathways. ECN may serve as a promising alternative adjuvant therapeutic agent for NSCLC patients diagnosed with metastatic cancers. It could reduce the invasive phenotypes of both low-invasive NSCLC cells at the primary tumor site and high-invasive NSCLC cells at metastatic locations in the lungs. Therefore, ECN treatment might help prevent cancer from spreading to other parts of the lungs or the body. While our findings may also inform future drug development efforts, the current research is not intended to establish ECN as a new synthetic drug candidate. Further studies employing animal models are necessary to validate these findings in vivo. Such studies will provide critical information on the pharmacokinetics, efficacy, and safety profile of ECN, whether used alone or in combination with other chemotherapy or adjuvant therapy, before conducting clinical trials. Finally, future research is essential for translating these results toward clinical application, leading to a novel treatment option for patients with metastatic NSCLC.

## 4. Materials and Methods

### 4.1. Chemicals, Reagents, and Antibodies

Eurycomalactone (ECL) and eurycomanone (ECN) were commercially purchased from Chengdu Biopurify Phytochemicals Ltd. (Chengdu, China) and were dissolved in dimethyl sulfoxide (DMSO) in all experiments. Dulbecco’s Modified Eagle Medium (DMEM), Roswell Park Memorial Institute (RPMI)-1640 medium, penicillin/streptomycin antibiotics, and trypsin-ethylenediaminetetraacetic acid (trypsin-EDTA) were purchased from Thermo Fisher Scientific, Inc. (Waltham, MA, USA). Fetal bovine serum (FBS) was obtained from GE Healthcare (Chicago, IL, USA). MTT was obtained from AppliChem GmbH (Darmstadt, Germany). TGF-β1 was obtained from PeproTech (Cranbury, NJ, USA). Mammalian protein extraction buffer was obtained from GE Healthcare (Chicago, IL, USA). Antibodies specific to E-cadherin and N-cadherin were purchased from Abcam (Cambridge, UK). Antibodies against vimentin, p-Smad2^(S465/467)^/Smad3^(S423/425)^, p^(S473)^-Akt, and Akt were purchased from Cell Signaling Technology (Danvers, MA, USA). Antibodies against Smad and β-actin were purchased from BD Biosciences (San Jose, CA, USA) and Sigma-Aldrich (St. Louis, MO, USA), respectively. Horseradish peroxidase (HRP)-conjugated goat anti-rabbit or goat anti-mouse IgG was purchased from Bio-Rad Laboratories, Inc. (Hercules, CA, USA).

### 4.2. Cell Lines and Cell Cultures

The human NSCLC cell lines, A549 and Calu-1, were obtained from the American Type Culture Collection (ATCC, Manassas, VA, USA) and the CLS Cell Lines Service GmbH (Eppelheim, Germany), respectively. A549 cells were cultured in DMEM and Calu-1 cells in RPMI medium, which were supplemented with 10% fetal bovine serum (FBS), 1% glutamine, and 1% penicillin–streptomycin at 37 °C in a humidified atmosphere containing 5% CO_2_. The cell confluence at 80–90% was harvested and used for further experiments.

### 4.3. Cytotoxicity Test by MTT Assay

A549 (1.5 × 10^4^) or Calu-1 (2 × 10^4^) cells were seeded in a 96-well plate and cultured for 24 h. Cells were tested with various concentrations of ECL (0–20 µM) or ECN (0–30 µM) in serum-free media for 24 and 48 h. Next, the medium was removed before MTT dye solution was added to each well and incubated at 37 °C for four hours. Finally, all solutions were removed, and DMSO was added to dissolve the formazan crystal. The optical density of the violet solution was measured at 540 and 630 nm using a microplate reader (BioTek, Winooski, VT, USA). The percentage of cell viability was calculated as follows: % Cell viability (OD_540–630_ treated/OD_540–630_ control) × 100%. The non-toxic concentrations are considered as the concentrations that are lower or equal to the inhibitory concentration of 20% (≤IC20) of ECL and ECN on NSCLC cell viability and selected for further experiments.

### 4.4. Wound-Healing Assay

NSCLC cells were cultured in six-well plates until they reached about 90–100% confluence. Next, one linear wound was scraped into each well and washed with PBS to remove cellular debris. Cells were co-treated with or without TGF-β1 (10 ng/mL) and the non-toxic doses of ECL or ECN in serum-free media for 24 or 48 h. Images of the wound area were captured at 0, 24, and 48 h under an inverted microscope. The gap area was determined using the ImageJ software (ver. 1.53; National Institutes of Health, Bethesda, MD, USA) and the migration percentage was calculated as follows: % Migration = (A_0_ − A_T_/A_0_) × 100 where A_0_ is the gap area measured at hour 0 and A_T_ is the gap area at the indicated time (T).

### 4.5. Gelatin Zymography Assay

The conditioned media of A549 or Calu-1 cells were collected after the co-treatment of the non-toxic doses of test compound with or without TGF-β1 (10 ng/mL), and then the protein concentration was determined by Bradford assay. An equal amount of protein was separated on an 8% SDS-PAGE gel containing 0.1% (*w*/*v*) gelatin under nonreducing conditions for two hours. After electrophoresis, gels were washed in a solution containing 2.5% (*v*/*v*) Triton X-100. Then the gels were incubated in an enzymatic activation buffer for 24 h at 37 °C. After incubation, gels were stained with 0.5% (*w*/*v*) Coomassie blue R-250 solution and further incubated in a de-staining buffer. Zones of gelatinolytic activity, representing the level of MMP-2 secretion, were detected as a clear band against a blue background.

### 4.6. Transwell Migration and Invasion Assay

A549 or Calu-1 (2 × 10^4^ cells) suspended in serum-free media with or without TGF-β1 (10 ng/mL) and ECN were placed into the upper compartment of the transwell chamber and incubated for 12, 24, or 48 h. The medium of 5% FBS was used as a chemoattractant and filled into the lower compartment. The invasive cells were fixed with 100% EtOH for five minutes and stained with 0.5% crystal violet in 20% MeOH for 30 min. Finally, five random fields in each treatment were selected. The number of invasive cells was counted, and the migration or invasion percentage was calculated.

Transwell invasion assay was performed using the same procedure as the transwell migration assay with the transwell chamber precoated with Matrigel (Corning, NY, USA) and solidified in a 37 °C incubator for one hour to form a thin gel layer.

### 4.7. Western Blotting

Protein expression levels of E-cadherin, N-cadherin, vimentin, p-Smad2, Smad2, p-Akt, Akt, and β-actin were determined by immunoblotting. The amount of protein of A549 or Calu-1 cells after the co-treatment of ECN with or without TGF-β1 (10 ng/mL) was separated in 10% SDS-PAGE. The separated proteins were transferred to a nitrocellulose membrane. The target proteins were probed with specific primary antibodies at 4 °C overnight, followed by HRP-conjugated secondary antibodies for two hours at room temperature. Finally, the target protein band(s) were detected by chemiluminescence.

### 4.8. Statistical Analysis

All values are given as mean ± standard deviation (X ± SD) from triplicate samples of three independent experiments. Overall differences among the treatment groups were determined using a T-test or a one-way analysis of variance (ANOVA), followed by the Tukey test by GraphPad Prism version 10.4.2, GraphPad Software, Boston, MA, USA. *p*-values < 0.05 are regarded as significant.

## Figures and Tables

**Table 1:** The 20% and 50% Inhibitory Concentration (IC20 and IC50) of ECL and ECN on Human NSCLC A549 and Calu-1 Cells.

Cell Lines	Types of Cells	Eurycomalactone (ECL)				Eurycomanone (ECN)			
		IC20 (μM)		IC50 (μM)		IC20 (μM)		IC50 (μM)	
		24 h	48 h	24 h	48 h	24 h	48 h	24 h	48 h
A549	Adenocarcinoma	1.29 ± 0.27	0.87 ± 0.16	>20	2.61 ± 0.64	25.31 ± 12.51	14.77 ± 2.98	>30	>30
Calu-1	Squamous cell carcinoma	2.86 ± 0.74	1.90 ± 0.47	>20	4.84 ± 0.43	15.86 ± 4.74	20.84 ± 2.33	>30	>30

## Data Availability

The datasets used and/or analyzed during the current study are available from the corresponding author upon reasonable request.
